# Implementation of an Integrated Pediatric Perioperative Pain Pathway: A Quality Improvement Initiative

**DOI:** 10.1155/anrp/8014510

**Published:** 2025-03-27

**Authors:** Brynn P. Charron, Niveditha Karuppiah, Ushma Shah, Ryan Katchky, Raju Poolacherla

**Affiliations:** ^1^Schulich School of Medicine & Dentistry, Western University, London, Ontario, Canada; ^2^London Health Sciences Centre, London, Ontario, Canada; ^3^Faculty of Health Sciences, McMaster University, Hamilton, Ontario, Canada; ^4^Hamilton Health Sciences, Hamilton, Ontario, Canada; ^5^Scarborough Health Network, Scarborough, Ontario, Canada

## Abstract

**Purpose:** A quality improvement initiative was developed, implemented, and evaluated to improve pediatric postsurgical pain management and reduce hospital length of stay.

**Methods:** An interdisciplinary working group developed the novel integrated pediatric perioperative pain (IP3) pathway adhering to the 3P approach to pain management. Preoperative psychological intervention, patient and caregiver education, standardized medication ordersets, and early identification of increased pain were the focus. Length of stay, opioid consumption, and achievement of physiotherapy goals were compared pre- and postpathway for children undergoing posterior spinal instrumentation and fusion (PSIF) for adolescent idiopathic scoliosis (AIS).

**Results:** The prepathway (*n* = 34) and postpathway (*n* = 29) groups were well matched for age, gender, weight, number of levels fused, and socioeconomic status. Postpathway, length of stay in the intensive care unit (pre 1.06 days and post 0.76 days, *p* ≤ 0.01) and length of hospital stay (pre 6.24 days and post 5.11 days, *p* ≤ 0.01) significantly decreased. Standardized physiotherapy goals were achieved earlier postpathway implementation. Day of surgery postoperative opioid consumption was reduced in the postpathway group.

**Discussion:** Implementation of the IP3 pathway resulted in significant improvement in pain management for children undergoing major orthopedic surgery. Shortened hospital stay, earlier achievement of physiotherapy goals, and reduced initial opioid consumption were realized. Future efforts will focus on applying this pathway to other pediatric surgeries, optimizing surgical scheduling, and enhancing staff education.

**Summary**

• A novel integrated pediatric perioperative pain pathway was developed, implemented, and evaluated to improve pediatric postsurgical pain management and reduce hospital length of stay.
• Preoperative psychological intervention, patient and caregiver education, standardized medication ordersets, and early identification of increased pain were the focus.
• Shortened hospital stay, earlier achievement of physiotherapy goals, and reduced initial opioid consumption were realized.

## 1. Introduction

Chronic postsurgical pain is a common postoperative complication, impacting 20%–50% of children undergoing major surgery [1]. Perioperative pain management following major surgery is essential in preventing chronic postsurgical pain. Opioids have traditionally been used for pain control for pediatric patients undergoing major surgeries [2]. With a goal of decreasing opioid consumption and opioid-related adverse effects, the use of multimodal analgesia (MMA) has been explored [3, 4]. MMA has been investigated in adult orthopedic procedures, with evidence indicating that these diverse pain management protocols reduce opioid consumption, improve pain control, and shorten hospital stays [5]. Comprehensive literature reviews support the use of MMA in adult spine surgeries; however, additional investigation into pain management in pediatric spine surgeries is needed [6, 7].

The effectiveness of different MMA models has been studied in relation to pain control and their ability to support enhanced recovery after surgery (ERAS) protocols [8–10]. It was found that the inclusion of an MMA model was a critically important element in the success of these protocols [11, 12]. ERAS protocols were developed to accelerate functional recovery; decrease pain, complication rates, and length of stay; and subsequently increase patient satisfaction while decreasing healthcare costs. The inclusion of preoperative education, MMA models, early mobilization, timely transition to oral medications, prompt removal of drains/catheters, return to regular diet, monitoring of patient pain scores, and involvement of multidisciplinary teams are all components of ERAS protocols.

Posterior spinal instrumentation and fusion (PSIF) involve significant pain due to extensive dissection, inflammation, and central and peripheral nerve sensitization [13]. Pediatric spine surgeries are commonly associated with higher rates of chronic pain and longer postoperative recovery [14]. Chronic postsurgical pain is associated with prolongation of recovery, higher risk of postsurgical infection, and psychological distress for the child and their caregivers, leading to missed school days and higher healthcare costs [15]. Many factors have been identified as risk factors for chronic postsurgical pain such as type of surgery, acute postoperative pain, and various psychosocial factors [16, 17].

Prior to the implementation of the integrated pediatric perioperative pain (IP3) pathway at our institution, postoperative pain following major pediatric surgery was managed by a mixed (adult and pediatric) acute pain service. A retrospective audit of pediatric patients undergoing major orthopedic procedures examining opioid consumption, length of stay in the intensive care unit (ICU), length of stay in hospital, reported pain scores, and physiotherapy goal achievement was undertaken. Although the study encompassed children undergoing major surgeries, emphasis was placed on those undergoing PSIF for adolescent idiopathic scoliosis (AIS). Using the Institute for Healthcare Improvement's *Model for Improvement* framework, multiple Plan-Do-Study-Act (PDSA) cycles were completed. These focused on preoperative psychological interventions, education for patients and caregivers, standardized medication order sets, and early detection of increased pain levels. The IP3 pathway was structured to facilitate a multidisciplinary approach to support children and their families undergoing major surgery. Specifically with regard to PSIF for AIS, children and their families met with a physiotherapist, anesthetist, clinical nurse, and social worker ahead of surgery to discuss plans for the day of surgery and postoperatively what to expect. Also, standard ordersets were implemented based off of weight-based dosing of acetaminophen, ibuprofen, and morphine for all patients assuming no contraindications. One important change was to ensure that acetaminophen and ibuprofen were ordered as scheduled medications rather than as-needed medications. Children who could manage a patient-controlled analgesia pump were provided with this as well. The newly formed Pediatric Acute Pain Service directed the pain management strategies following the IP3 pathway implementation. The aim of this QI initiative was to (1) create, implement, and assess an innovative IP3 pathway, with the goal of enhancing perioperative pain management for children undergoing major orthopedic surgical procedures, (2) model a sustainable interdisciplinary team and obtain permanent funding, and (3) provide continuing education to healthcare professionals on pediatric pain management within and outside the organization.

## 2. Methods

A retrospective chart review of patients under 18 years of age who underwent PSIF for AIS from January 1, 2018, to December 31, 2019, was completed. This project was classified as a QI investigation based on the requirements listed in the Tri-Council Policy Statement, and thus ethics approval was waived by local research ethics board. Patient confidentiality was protected throughout, and all data analyzed were deidentified. Relevant charts were identified by codes pertaining to PSIF from operative lists within the designated time frame. Exclusion criteria included combined nonspine procedures and spinal fusion for reasons other than AIS. Categories of data collection were basic demographics, analgesics, pain assessment, physiotherapy achievements, and psychosocial and postoperative complications. Opioid consumption was converted into morphine equivalents per kilogram. Pain scores were recorded as numerical values on a scale of 0–10. Physiotherapy goals that were assessed and collected from patient charts included postoperative day when the patient sat on the edge of the bed, walked to the bathroom with minimal assistance, tolerated a seated position for 30 min, and completed 3 steps up and down stairs. These physiotherapy goals align with discharge criteria. Charts were reviewed to determine if these patients also accessed psychosocial support during the perioperative period (social worker, psychologist, etc.).

Following root cause analysis (Supporting Figure 1), the novel IP3 pathway was developed and implemented with a focus on perioperative pain education (Supporting Figure 2). The initiative was started in 2020; however, due to the COVID-19 pandemic, intermittent pauses of change implementation and analysis were encountered. The IP3 pathway integrated services through a multidisciplinary approach. PDSA cycles focused on the standardization of prescription practices, timely involvement of physiotherapy, early engagement of child life and social work support, and psychological intervention for patients at risk for chronic pain, chronic opioid use, anxiety, or depression were created. Balancing measures included nursing personnel to run the preoperative clinic and time away from clinical duties for clinic-based healthcare workers. Initially, individual funding sources were required to support pathway personnel; however, permanent funding through the hospital was then obtained for continued implementation. Microsoft Excel was used for data analysis. Frequency and descriptive statistics were completed with statistical significance defined as *p* value less than 0.1.

## 3. Results

Prior to pathway implementation, 34 cases of individuals undergoing PSIF for AIS were identified as relevant from the selected time period for this retrospective review. Following pathway implementation, 34 cases of individuals undergoing PSIF for AIS were identified; however, five of these children did not have adequate documentation of pain medications administered postoperatively resulting in exclusion from analysis.

Pre- and postpathway groups were well-matched for age, gender, weight, mean number of levels fused, and socioeconomic status (Supporting Table 1). Socioeconomic status was determined by the Statistics Canada Geomapping which considered the average household income, household education level, and housing value for the inputted postal code [18].

### 3.1. Analgesia

**Figure 1:**
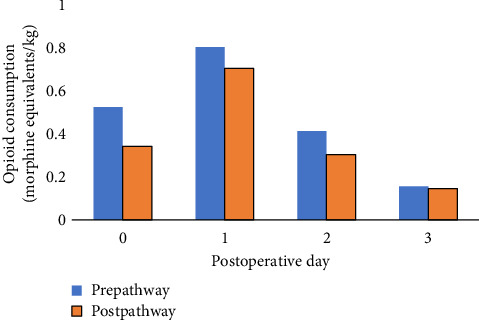
Postoperative opioid consumption per day in the pre- and postpathway groups.

Analysis pre- and postpathway demonstrated that all patients received opioids postoperatively. Postoperatively, patients were provided morphine and hydromorphone, through oral, intravenous, and/or subcutaneous dosing. All patients received postoperative opioid analgesic medication through either patient-controlled analgesic (PCA) pumps or continuous infusions. In both pre- and postpathway groups, the highest average amount of morphine equivalents was administered on POD 1. The average morphine equivalents were decreased in the postpathway as compared to prepathway on all postoperative days with the difference on POD 0 and 2 being significant (Figure 1, Supporting Table 2).

In the prepathway group, acetaminophen and NSAIDs were not uniformly administered and not all patients received scheduled medications, whereas postpathway, all patients received scheduled acetaminophen and NSAIDs. A portion of patients in the pre- and postpathway groups received adjuvant medications including gabapentin, baclofen, diazepam, lorazepam, pregabalin, and amitriptyline. Discontinuation of intravenous medications most frequently occurred on POD 2.

### 3.2. Pain Assessment

Prepathway pain scores were not documented consistently with some records having missing data points. Postpathway maximum pain scores were recorded for POD 0–2 in a more consistent manner. Given the inconsistent reporting of pain scores prepathway implementation, a direct comparison of this metric was limited. The general trend showed an average decrease in maximum pain scores from POD 0–2 when comparing postpathway to prepathway recordings (Supporting Table 3).

### 3.3. Length of Stay

The average ICU and overall hospital stay lengths were significantly shortened in the postpathway group (mean ICU stay: prepathway 1.06 ± 0.06 days and postpathway 0.76 ± 0.08 days, *p* < 0.01, and mean hospital stay: prepathway 6.24 ± 0.19 days and postpathway 5.11 ± 0.14 days, *p* < 0.01). At our institution, PSIF for AIS are typically performed on Tuesdays which was represented in the pre- and postpathway groups with 91% and 97% occurring on Tuesdays, respectively.

### 3.4. Postoperative Physiotherapy

The day of achievement for nearly all physiotherapy goals was significantly earlier following pathway implementation (Supporting Table 4). Prepathway, postoperative physiotherapy was received by all patients who underwent PSIF for AIS; however, the timing of the commencement of physiotherapy was inconsistent due to the orders being placed at different times after surgery and by different healthcare staff. Postpathway, postoperative physiotherapy orders were standardized and initiated with basic postoperative order sets. Postpathway, all patients were seen by physiotherapy on post-op day 1 regardless of the day of the week that the surgery was performed.

### 3.5. Post-Discharge Course

Prior to the implementation of the IP3 pathway, referral to pain services including pain management, psychiatry, social work, and physiotherapy was completed on a case-by-case basis. Prepathway, two children who underwent PSIF for AIS had continuing pain 2 years postsurgery and were rereferred to the pediatric orthopedic department for reassessment. From the available documentation, neither of these patients were provided a referral for chronic pain assessments. Following IP3 pathway implementation, a standardized protocol after major pediatric surgery triggered a multidisciplinary team approach. All patients undergoing PSIF for AIS were assessed by a pain specialist, clinical nurse, social worker/psychologist, and physiotherapist in addition to the standard practice of preoperative anesthesia and surgical consultations. Postpathway implementation, one patient was noted to have ongoing pain resulting in difficulty with daily function beyond three months post-op. Hardware irritation occurred necessitating revision surgery. Following revision surgery, referral to a pediatric chronic pain program was discussed with the family; however, based upon the available documentation, this was not accessed through this institution's Pediatric Chronic Pain Program but may have been sought at another institution.

Of the patients in the IP3 pathway, the majority were discharged from pain service care following the 2-week post-op assessment. Five children were lost to follow-up after discharge from hospital, and none were rereferred to the pain service by the treating orthopedic surgeon. Of the patients assessed in the IP3 pathway at the 3-month post-op assessment, the Quality of Recovery-15 Score was calculated. The Quality of Recovery Score ranges from 0 to 150 with four scoring levels as follows: excellent—136–150, good—122–135, moderate—90–121, and poor—0–89. Of the patients assessed at three months post-op, 44.4% were in the excellent range, 33.3% were in the good range, and 22.2% were in the moderate range. Informal impressions put forth by family members and patients indicated general satisfaction with the IP3 pathway. One suggestion was for increased written documentation.

## 4. Discussion

A prepathway audit of the pediatric pain management strategies that were in place at this tertiary care center was conducted. Given the longer than global average length of stay for patients undergoing PSIF for AIS, this patient population was focused on. The IP3 pathway serves as a prototype and will be adopted for other major pediatric surgeries at our institution. The pathway provides integration of services through a multidisciplinary approach to the management of pain. Standardization of analgesic prescribing practices was addressed. Timely involvement of physiotherapy, child life, social work, and psychological intervention for at-risk patients was incorporated. Issues such as extended opioid use, chronic pain, anxiety, and depression are areas of particular focus with regard to pediatric patients undergoing major surgeries.

The prepathway audit revealed significant variability in pain management strategies used in the pediatric patient population at our institution. Lack of standardization was thought to contribute to increased use of opioids during admission, longer time to reach discharge milestones, increased length of hospital stay, and higher reported pain scores. Previous studies assessing the recovery of pediatric patients from major surgeries indicated that while NSAIDs, acetaminophen, and other adjuvant medications alone may provide benefit, administering MMA regimens should be considered to optimize pain management [10, 19]. Our prepathway analyses indicated that administration of oral NSAIDs, acetaminophen, and other adjuvant medications occurred with considerable variability. Prior to the pathway, only 88% of patients received scheduled acetaminophen and 53% received scheduled NSAIDs. Implementation of the IP3 pathway resulted in all patients having standardized dosing and scheduling of acetaminophen and NSAIDs. Pain scores were also a studied metric and found following postpathway implementation to be more consistently recorded as compared to prepathway. There was a general trend postpathway implementation that the maximum pain scores on POD 0–2 were lower as compared to prepathway. Staff education regarding the importance of accurate and uniform postoperative pain scores was highlighted through this study. Earlier achievement of physiotherapy goals and in turn shorter hospital stay duration were shown among the postpathway patients. Education obtained preoperatively regarding postoperative pain expectations, standardized medication protocols, and early physiotherapy participation contributed to improved pain management postpathway implementation as seen with earlier discharge to home.

Studies investigating ERAS protocols highlighted the importance of a multidisciplinary approach, involving clinicians and patient stakeholders, to accelerate recovery and facilitate early discharge [20, 21]. A review focused on electrophysiology studies and ablation procedures among pediatric patients highlighted the importance of carefully selecting medications based off of side effects and efficacy. The proposed protocol by Monaco et al. incorporated preoperative evaluations and education in addition to multiple points of intervention on the day of procedure [22]. This mirrored the IP3 pathway in the recognized key time points for impacting patients' postoperative course. A meta-analysis of ERAS in AIS patients observed that all studies reviewed included the integration of multidisciplinary teams in patient care [23, 24]. Prepathway, patients' care involved various healthcare team members inclusive of surgeons, acute pain services, physiotherapists, child life experts, and social workers; however, without direct communication between team members consistent, standardized care was not provided. Furthermore, Ursoleo et al. highlighted the importance of promoting communication and education within the patient–physician relationship to empower patients and their families in shared decision making [25]. The development of the IP3 pathway was beneficial as it improved workflow, facilitated patient care, and decreased system costs through shortened length of ICU and hospital stays.

Discharge planning encompasses multiple points in the care timeline of patients undergoing PSIF, including preoperative planning and patient education [26]. This includes scheduling surgeries on a day of the week that allows healthcare team members to complete assessments and provide care within a timeframe that facilitates presurgical education. At our institution, PSIF for AIS is generally completed on a consistent day of the week. Ensuring translation of achievement of physiotherapy goal to discharge readiness is crucial. Review of timing of physiotherapy discharge milestones and length of hospital stay should be considered to confirm that patient discharge is not delayed due to weekend staffing. Patient education regarding the ERAS protocols and realistic patient expectations are shown to accelerate recovery [27, 28]. Postpathway implementation, both patients and their families reported overall satisfaction with the multidisciplinary approach and preoperative education.

The prepathway portion of the study was limited by its retrospective design. There was no standardized recording of data which presented some challenges when comparing pre- and postpathway metrics. Pain scores specifically were inconsistently recorded prepathway limiting conclusions when compared to postpathway data. Prepathway patients had surgery prior to the COVID-19 pandemic. Although the impact of COVID-19 on patients undergoing surgery after pathway implementation is impossible to quantify, patients likely encountered rescheduling of appointments/surgeries contributing to heightened anxiety. Conclusions are constrained by the small number of studied patients. Further improvement efforts are required to refine patient selection and organize operative bookings to maximize time for preoperative interventions. Recently, there has been substantial focus on induction agents and correspondence to postoperative pain [29]. Additional incorporation of this aspect into the IP3 is a future direction given that only postoperative pharmaceutical regimens were standardized. The expansion of the IP3 pathway to include other pediatric surgeries is currently underway.

## 5. Conclusions

The development and implementation of a novel IP3 pathway based off the 3P approach was successfully incorporated at our institution. This quality improvement initiative aimed to address preoperative patient and caregiver education, standardization of prescribing practices, and postoperative pain management. A key aspect of the IP3 pathway was the involvement and coordination of a multidisciplinary team. Following pathway implementation, postoperative opioid consumption was reduced, physiotherapy milestones were achieved earlier, and length of stays were shortened. The utilization of the IP3 pathway framework can be expanded to other pediatric surgeries both at our institution and other healthcare facilities.

## Data Availability

Data are available through contacting the corresponding author.

## Supporting Information

As part of the supporting information, readers can view the Ishikawa diagram demonstrating the basis of the initiative as well as the process map representing the ideal state of the integrated pediatric perioperative pain pathway (IP3). In the provided tables, data pertaining to the demographic information of patients, pre- and postpathway postoperative analgesic requirements, pre- and postpathway postoperative maximum daily pain scores, and pre- and postpathway postoperative achievement of physiotherapy goals are summarized.

A novel integrated pediatric perioperative pain pathway was developed, implemented, and evaluated to improve pediatric postsurgical pain management and reduce hospital length of stay. Preoperative psychological intervention, patient and caregiver education, standardized medication ordersets, and early identification of increased pain were the focus. Shortened hospital stay, earlier achievement of physiotherapy goals, and reduced initial opioid consumption were realized.

Supporting Figure 1—Ishikawa diagram demonstrating root cause analysis for the QI initiative.

Supporting Figure 2—process map representing ideal state of the integrated pediatric perioperative pain pathway (IP3).

Supporting Table 1—pre- and postpathway demographic information of included cases of individuals who underwent PSIF for AIS.

Supporting Table 2—pre- and postpathway postoperative analgesic requirements including total daily opioid consumption.

Supporting Table 3—pre- and postpathway postoperative maximum daily pain scores on 0 to 10 pain scale.

Supporting Table 4—pre- and postpathway postoperative achievement of physiotherapy goals.
