# Supplementary material for: Implementation of an Integrated Pediatric Perioperative Pain Pathway: A Quality Improvement Initiative
Source: Anesthesiol Res Pract. 2025 Mar 27;2025:8014510. doi: 10.1155/anrp/8014510 (PMC11968164; doi:10.1155/anrp/8014510)
Supplement: Supporting Information — Additional supporting information can be found online in the Supporting Information section. [file 8014510.f1.docx]

**Supplementary Information**


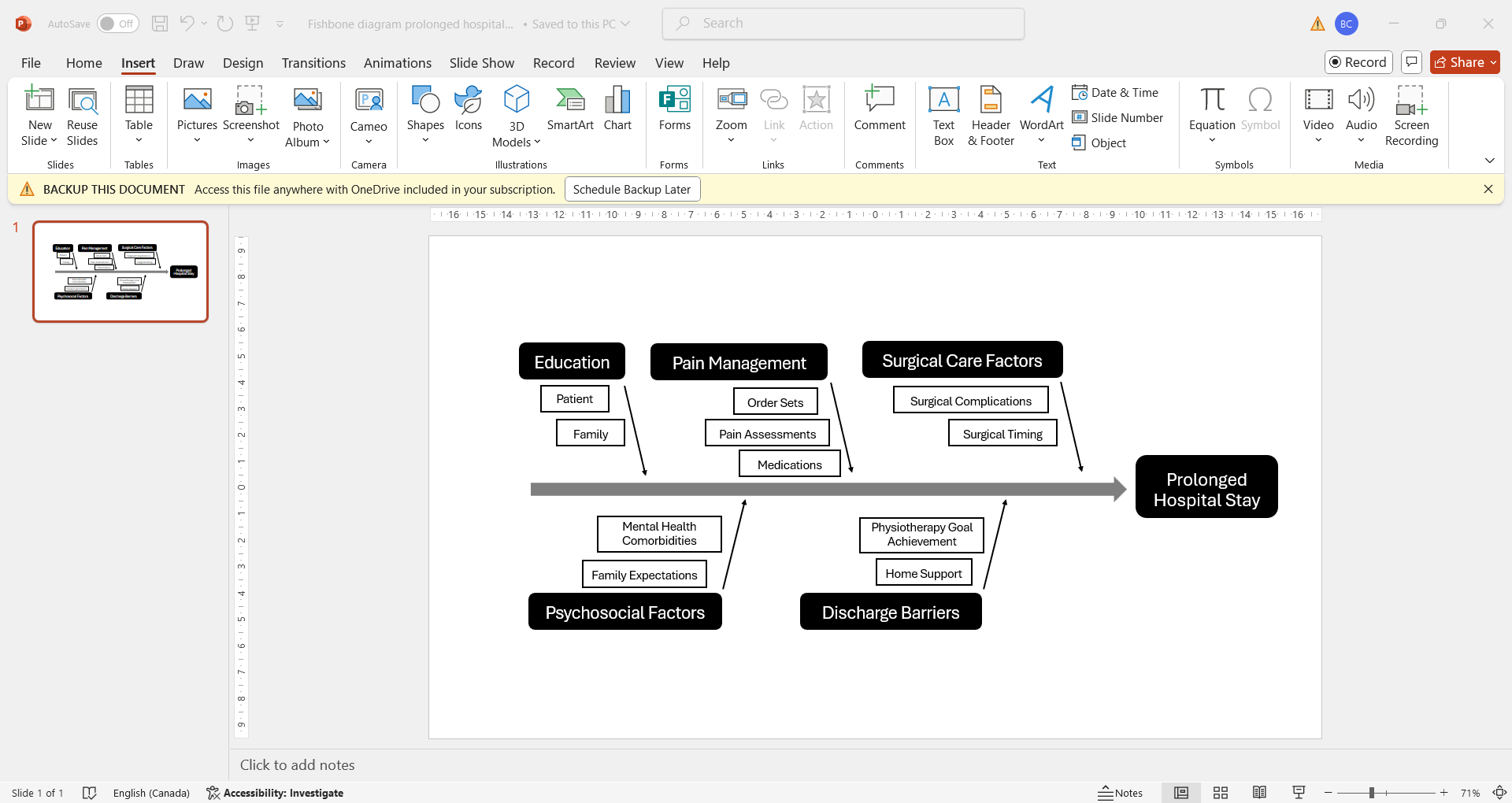


Supplemental Figure 1 – Ishikawa diagram demonstrating root cause analysis for the QI initiative.


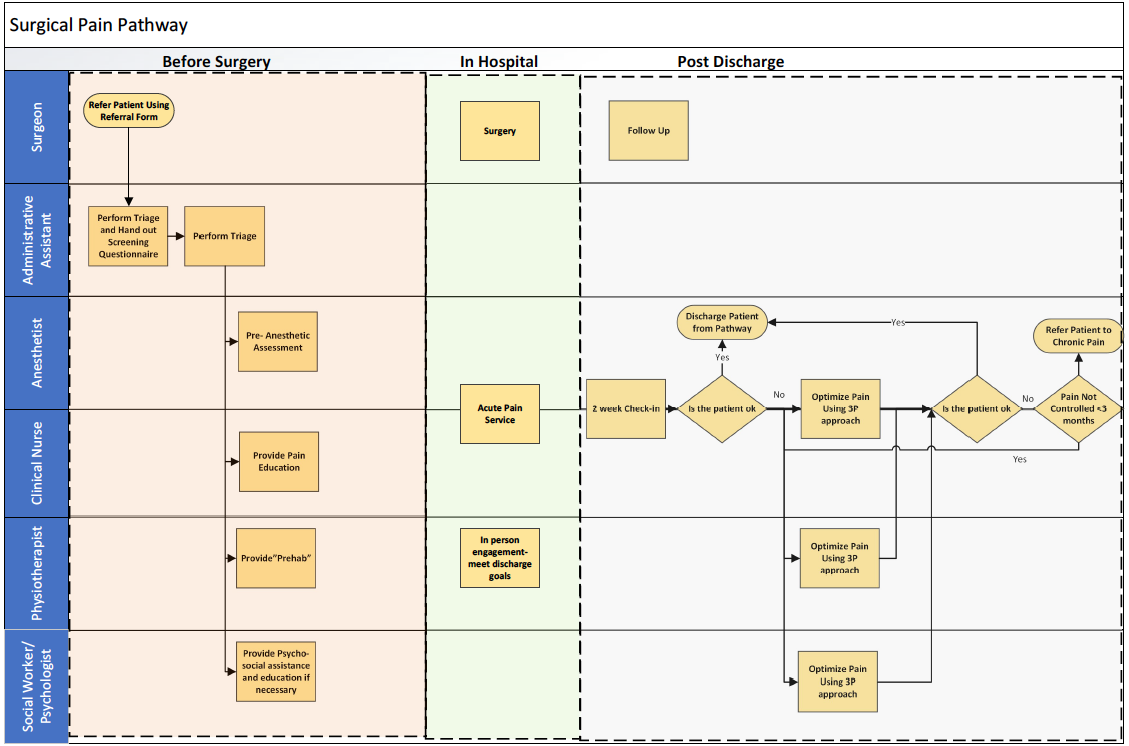


Supplemental Figure 2 – Process map representing ideal state of the Integrated Paediatric Perioperative Pain Pathway (IP3).

Supplemental Table 1. Pre- and post-pathway demographic information of included cases of individuals who underwent PSIF for AIS.

|  | Pre Pathway (n=34) | Post Pathway (n=29) | p value |
| --- | --- | --- | --- |
| Mean Age (years) +/- SE | 14.12 +/- 0.32 | 14.66 +/- 0.34 | 0.13 |
| Females (%) | 82.4 | 79.3 | 0.38 |
| Weight (kg) +/- SE | 55.0 +/- 1.92 | 57.4 +/- 3.04 | 0.26 |
| Mean Number of Levels Fused +/- SE | 11.6 +/- 0.21 | 11.1 +/- 0.31 | 0.12 |
| Socioeconomic Status (Z score) +/- SE | -1.43 +/- 0.40 | -1.26 +/- 0.50 | 0.40 |

Supplemental Table 2. Pre- and post-pathway postoperative analgesic requirements including total daily opioid consumption.

|  | Pre Pathway (n=34) | Post Pathway (n=29) | p value |
| --- | --- | --- | --- |
| Average Post-Op Day  PCA Discontinued (Day) +/- SE | 2.09 +/- 0.11 | 1.97 +/- 0.09 | 0.20 |
| Average Morphine Equivalents | | | |
| POD 0 (mg/kg) +/- SE | 0.52 +/- 0.07 | 0.34 +/- 0.04 | ≤ 0.01 |
| POD 1 (mg/kg) +/-SE | 0.80 +/- 0.08 | 0.71 +/- 0.10 | 0.23 |
| POD 2 (mg/kg) +/- SE | 0.41 +/- 0.06 | 0.30 +/- 0.05 | 0.09 |
| POD 3 (mg/kg) +/- SE | 0.16 +/- 0.03 | 0.14 +/- 0.02 | 0.37 |
| Scheduled oral Acetaminophen (%) | 88.3 | 100 |  |
| Scheduled oral NSAIDs (%) | 53.6 | 100 |  |
| Adjuvant medications (%) | 33.4 | 31.8 |  |

Supplemental Table 3 **-** Pre- and post-pathway postoperative maximum daily pain scores on 0 to 10 pain scale.

| Maximum Score (1-10 scale) +/- SE | Pre-Pathway (n=34) | Post-Pathway (n=29) | p value |
| --- | --- | --- | --- |
| POD 0 | 7.44 +/- 0.34 | 7.24 +/- 0.34 | 0.34 |
| POD 1 | 6.94 +/- 0.36 | 5.47 +/- 0.44 | ≤ 0.01 |
| POD 2 | 6.05 +/- 0.31 | 5.24 +/- 0.50 | 0.09 |

Supplemental Table 4 - Pre- and post-pathway postoperative achievement of physiotherapy goals.

| Average Day Goal Met | Pre-Pathway (n=34) | Post-Pathway (n=29) | p value |
| --- | --- | --- | --- |
| End of Bed Sit (day) +/- SE | 1.15 +/- 0.06 | 1.24 +/- 0.11 | 0.23 |
| > 30 minutes chair sit (day) +/- SE | 2.94 +/- 0.18 | 2.41 +/- 0.14 | ≤ 0.01 |
| Ambulating > 5m (day) +/-SE | 3.35 +/- 0.12 | 2.93 +/- 0.08 | ≤ 0.01 |
| Up and Down 3 stairs (day) +/- SE | 4.53 +/- 0.17 | 3.97 +/- 0.12 | ≤ 0.01 |
